# Evaluation of the Predatory Mite *Neoseiulus barkeri* against Spider Mites Damaging Rubber Trees

**DOI:** 10.3390/insects14070648

**Published:** 2023-07-18

**Authors:** Junyu Chen, Lijiu Zheng, Zhengpei Ye, Jianyun Wang, Fangping Zhang, Yueguan Fu, Chenghui Zhang

**Affiliations:** 1 College of Ecology and Environment, Hainan University, Haikou 570100, China; jychen@catas.cn (J.C.); zhenglijiu@163.com (L.Z.); 2 Environment and Plant Protection Institute, Chinese Academy of Tropical Agricultural Sciences, Haikou 570100, China; 3 Engineering Research Center for Biological Control of Tropical Crops Diseases and Insect Pest, Haikou 570100, China; 4 Key Laboratory of Integrated Pest Management on Tropical Crops, Ministry of Agriculture and Rural Affairs, Haikou 570100, China

**Keywords:** mite, development, fecundity, population growth, biological control

## Abstract

**Simple Summary:**

Phytoseiid mite *Neoseiulus barkeri* is a widely recognized and commercially accessible predator of many insects and pest mites, with a global presence. In this study, we evaluated the biological control potential of *N. barkeri* against *Eotetranychus sexmaculatus*, *Eutetranychus orientalis* and *Oligonychus biharensisin*, which are major spider mites causing serious damage to rubber trees in China. The biological performance of *N. barkeri* on these pests in comparison to that on *Tyrophagus putrescentiae*, a storage mite used to mass-rear this predator, was determined in the laboratory. When fed on these spider mites, *N. barkeri* could complete its life cycle and had a high fecundity on *E. orientalis* or *O. biharensisin*. It performed better on *E. orientalis* than on other two spider mites. It performed similarly on *O. biharensisin* and *T. putrescentiae*, in terms of its immature developmental period, survivorship, fecundity and intrinsic rate of increase on these preys. The data provides valuable insights into our understanding of the potential efficacy of *N. barkeri* as a biological control agent for the management of pest spider mites on rubber trees.

**Abstract:**

The spider mites *Eotetranychus sexmaculatus*, *Eutetranychus orientalis* and *Oligonychus biharensisin* are severe pests of rubber trees in China. The predatory mite *Neoseiulus barkeri* has been found to be a natural enemy of these three pests, while nothing is known about the biological performance of this phytoseiid predator against these phytophagous mites. In this study, the development, survivorship, reproduction, adult longevity, fecundity, sex ratio and population growth parameters of *N. barkeri* fed on these pests were evaluated in comparison to the factitious prey *Tyrophagus putrescentiae* in the laboratory at 25 ± 1 °C, 75 ± 5% relative humidity and a 12:12 (L:D) h photoperiod. The results showed that *N. barkeri* could develop from egg to adult and reproduced successfully on the three preys. The survival rate of *N. barkeri* from egg to adult was higher when fed on *E. orientalis* (100%) and *T. putrescentiae* (100%) than when fed on *O. biharensisin* (93.60%) and *E. sexmaculatus* (71.42%). The shortest and longest generation time for *N. barkeri* were observed on *E. orientalis* with 6.67 d and *E. sexmaculatus* with 12.50 d, respectively. The maximum fecundity (29.35 eggs per female) and highest intrinsic rate of increase (*r*_m_ = 0.226) were recorded when *N. barkeri* fed on *E. orientalis*, while feeding on *E. sexmaculatus* gave the minimum fecundity (1.87 eggs per female) and lowest reproduction rate (*r*_m_ = 0.041). The values of these parameters for *N. barkeri* evaluated on *O. biharensisin* were found to be comparable to those obtained on *T. putrescentiae*. The sex ratio of *N. barkeri* progeny on the preys mentioned above, apart from *O. biharensisin*, was female biased. According to the findings, *N. barkeri* could serve as a promising biocontrol agent against *E. orientalis* and *O. biharensisin*, and possibly *E. sexmaculatus* on rubber trees.

## 1. Introduction

Biological control is a cost-effective and environmentally friendly alternative to chemical control in managing pest [1]. Currently, there are more than 230 species of natural enemies available for augmentative biological control worldwide, with the Acari group, which includes 30 species of commercially available natural enemies, occupying the second largest group after hymenopteran parasitoids within the arthropods used in augmentative releases [2]. Predatory mites from the family Phytoseiidae have proven to be the most successful biocontrol agents from Acari used to control some of the most important pests including two-spotted spider mite *Tetranychus urticae*, western flower thrips *Frankliniella occidentalis* and whitefly *Bemisia tabaci*, which infest vegetables and ornamental crops worldwide [3,4,5]. For example, in 2005, the predatory mite *Amblyseius swirskii*, originating from the East Mediterranean coast, was introduced to the market, and by 2014, it had been sold to over 50 countries and used successfully in pest management in many greenhouse crops [3,6,7]. Most of the commercially used phytoseiid predatory mites belong to the genera *Amblyseius* and *Neoseiulus* [8]. As one of the earliest commercial biocontrol agents, *N. barkeri* has been available in the market for thrips control since 1981 [3,9]. Apart from thrips, it is now widely used to effectively control several other small insect pests and spider mites [10]. This predatory mite can be commonly found in citrus, mango, rubber and other plants, as well as stored products [11,12]. Due to its economic significance, the biological and ecological studies on *N. barkeri* have received a lot of attention. For instance, several studies have investigated the effects of different temperatures and photoperiods on the development and reproduction of *N. barkeri* [9,11,12,13], the impact of female age on mating and food deprivation periods on reproduction of *N. barkeri* [14], the effects of UV-B radiation on the survival and egg hatchability of *N. barkeri* [15] and the tolerance of *N. barkeri* to high temperature and desiccation stresses [16]. Although a limited number of studies have evaluated the biological performance of *N. barkeri* against phytophagous mites and pests of crops [9,17,18,19,20,21], little is known about its potential ability to control pests on trees. Further evaluation of the performance of *N. barkeri* against tree pests will provide a better understanding of its suitability as a biocontrol agent and potentially enhance its use in various fields.

**Figure 1:**
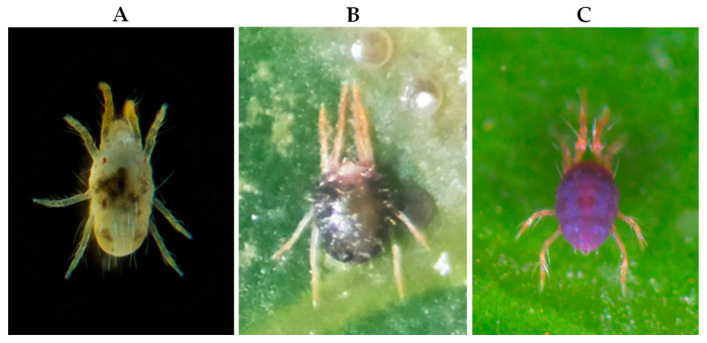
Three spider mites causing damage to rubber trees. (**A**) *Eotetranychus sexmaculatus*, (**B**) *Eutetranychus orientalis* and (**C**) *Oligonychus biharensisin*.

The rubber tree, *Hevea brasiliensis*, is originally from the Amazon, which is a highly valuable tree used in economical production of natural rubber latex [22]. Over recent decades, rubber plantations have expanded rapidly across southern China, particularly in Hainan Island, which possesses the largest rubber plantation area, accounting for approximately one quarter of the total flora [23]. Unfortunately, rubber trees are heavily damaging by a variety of phytophagous mites, including six-spotted mite *Eotetranychus sexmaculatus*, oriental red mite *Eutetranychus orientalis* and *Oligonychus biharensisin* in China (Figure 1) [24,25]. These three spider mites cannot spin web but seriously affect the growth and latex production of rubber trees, causing significant economic loss [26]. The control of these spider mites currently relies primarily on acaricides, but the overuse of acaricides has resulted in pesticide resistance among the spider mites, adverse effects on natural enemies, and environmental pollution [24,27]. Since *N. barkeri*, a commercially produced predatory mite, has been found to be a natural enemy of these phytophagous mites on the rubber tree in China [28,29], it has application potential for controlling them.

In this study, we assessed the biological performance of *N. barkeri* on *E. sexmaculatus*, *E. orientalis* and *O. biharensisin* in comparison to that on the mold mite *Tyrophagus putrescentiae*. This species is a common mite found ubiquitously in soil, stored products and house dust, which is often used as factitious prey for mass-rearing predatory mites, including *N. barkeri* [13,30]. The development, survival and fecundity of *N. barkeri* on all four preys were compared under laboratory conditions. The results provide valuable insights into the potential use of *N. barkeri* in an augmentative biological control program aimed at mitigating spider mite infestations in rubber trees.

## 2. Materials and Methods

### 2.1. Mites

A colony of the predatory mite *N. barkeri* used in this study was maintained in the laboratory for over 50 generations within artificial climate chambers. As a factitious prey, *T. putrescentiae* was utilized and fed with wheat bran for more than ten generations within an artificial climate box (MGC-300H, Blue pard, Shanghai, China) that originated from the laboratory’s stock culture. The preys *E. sexmaculatus*, *E. orientalis* and *O. biharensisin* (Figure 1) were collected from rubber trees located in the suburbs of Danzhou city, Hainan province, China, and maintained in the laboratory for more than five generations. These three spider mites were fed with leaves of rubber trees, which were kept in an upside-down position. To maintain leaf freshness, their host leaves from the middle stratum of the tree were placed on sterile water-absorbed cotton in a 25 cm × 18 cm disc. In line with their natural habits of damaging rubber tree leaves [26], leaf surface was placed upwards for rearing *E. sexmaculatus* and *E. orientalis*, while laid upside down for rearing *O. biharensisin*. Every 3 days, fresh rubber tree leaves were replaced. Predatory and prey mites were maintained at 25 ± 1 °C, 75 ± 5% relative humidity and a 12:12 (L:D) h photoperiod.

### 2.2. Experimental Unit

**Figure 2:**
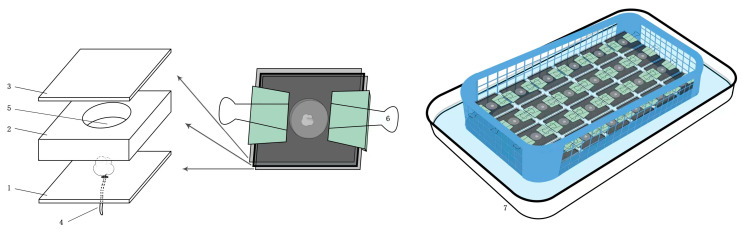
A schematic diagram of the experimental unit. 1, lower layer of the acrylic board; 2, middle layer of the acrylic board; 3, upper layer of the acrylic board; 4, cotton thread; 5, circular hole in the middle layer of the acrylic board (a space for mite activity); 6, long tail clamp; 7, enamel tray for containing water.

The experimental unit is a homemade structure designed to resemble a stock culture rearing unit (Figure 2), composing of three layers of acrylic board. The upper layer is 39.5 × 39.5 × 1.5 mm. The lower layer is 39.5 × 39.5 × 4 mm. The middle layer features a circular hole with a diameter of 18 mm in the center, which serves as a space for mite activity. To supply the moisture needed for the mites, a small hole with a diameter of 1.5 mm is positioned in the center of the lower layer. A cotton thread is threaded through this hole, which is dipped in water contained within an enamel tray. Water was added every day to prevent the cotton from drying out. The three-layer acrylic plates are secured together at the ends using long tail clamps, forming a closed feeding cell for mites.

### 2.3. Experimental Setup

The predatory mite *N. barkeri* was maintained in a rearing unit consisting of a Petri dish (5 cm diameter) with a foam plastic pad (3 cm diameter, 1 cm thick) soaked in water, which has a piece of filter paper (3 cm diameter) with plastic film (2 cm diameter) on it [31]. Each rearing unit housed an adult female *N. barkeri* with sufficient amounts of *E. sexmaculatus*, *E. orientalis, O. biharensisin* or *T. putrescentiae* provided as food. To ensure synchronized eggs for the experiments, newly laid *N. barkeri* eggs less than 8 h old were collected and transferred to the experimental unit using a fine camel hair brush. A single egg was placed in each experimental unit. Sixty eggs were tested in each biological replicate. Three biological replicates were conducted for each prey.

The hatching of *N. barkeri* eggs was observed every 8 h, at 7:00, 15:00 and 23:00 throughout the day. The hatchability and developmental period of the eggs were recorded. Once the eggs hatched, they were provided with an abundant supply of different types of prey as a food source. The development, duration of immature stages and mortality of *N. barkeri* were observed and recorded until they developed into adult mites. The newly emerged predatory adult mites were paired to obtain couples of one male and one female, which were subsequently placed into separate rearing units for individual cultivation. They were supplied with different adult preys as a food source. The longevity of the female predators and the number of eggs laid by them were recorded daily until their death. The eggs laid by the predators were collected and reared until adulthood, as described above, in order to determine the gender of their offspring. All experiments were carried out in an incubator under controlled conditions at 25 ± 1 °C, 75 ± 5% relative humidity, and a 12:12 (L:D) h photoperiod.

### 2.4. Data Analysis

Life table parameters of *N. barkeri* fed on various preys were determined by using the survivorship and fecundity, following the methods outlined by Xu [32]. The net increase rate *R_0_* is calculated as the sum of lx multiplied by m_x_. The mean generation time (*T*) was obtained as Σl_x_ × m_x_ × x/*R*_0_. The intrinsic rate of increase (*r*_m_) was computed using the formula *r*_m_= ln*R*_0_/*T*. The finite rate of increase (*λ*) was calculated as *λ* = e*^r^*_m_ and doubling time (DT) as DT = ln2/*r*_m_. In these equations, x represents a unit interval of time, l_x_ denotes the age-specific survival rate (proportion of individuals alive at age x), m_x_ connotes the age-specific fecundity (the number of female progenies per female at age x), and e is a constant of nature.

The data were prepared in Microsoft Excel and then subjected to statistical analysis using SPSS v.22.0 (SPSS, Chicago, IL, USA). The effects of different prey types on the development, mortality, longevity and fecundity of *N. barkeri* were analyzed using one-way ANOVA, and significant differences between means were confirmed by applying Tukey’s honestly significant difference (HSD) test. All the data were tested for the assumption of normality and homoscedasticity before ANOVA tests. The sex ratios of the offspring were compared using χ^2^ tests. The graphical illustrations were generated using Origin 7.5 (OriginLab Corporation, Northampton, MA, USA).

## 3. Results

### 3.1. Development Period of Immature Stages

The predatory mite *N. barkeri* completed its development successfully when fed on *E. sexmaculatus*, *E. orientalis*, *O. biharensisin* and *T. putrescentiae*. It showed no difference in the egg (*F* = 0.269; df1 = 3, df2 = 173; P = 0.848) and protonymph (*F* = 0.246; df1 = 3, df2 = 173; P = 0.864) developmental times when fed on the four different prey types (Table 1). However, the developmental duration of larvae (*F* = 18.869; df1 = 3, df2 = 173; P < 0.001), deutonymph (*F* = 2.827; df1 = 3, df2 = 173; P = 0.04) and total immature stage (*F* = 17.815; df1 = 3, df2 = 104; P < 0.001) of *N. barkeri* were significantly influenced by preys. When *E. sexmaculatus* was used as a food source, the development time of larval *N. barkeri* was the slowest at 1.52 ± 0.11 d. In contrast, *N. barkeri* developed most rapidly when fed on *E. orientalis*, taking only 0.67 ± 0.03 d to complete the larval stage. Developmental period for larvae fed on *O. biharensisin* and *T. putrescentiae* did not significantly differ (*F* = 2.119; df1 = 1, df2 = 69; P = 0.150). The deutonymph developmental duration of *N. barkeri* was observed to be significantly longer when fed on *T. putrescentiae* compared to those fed the other three preys (F = 5.319; df1 = 3, df2 = 156; P = 0.002). The shortest and longest generation time recorded for *N. barkeri* were 6.67 ± 0.08 d and 12.50 ± 0.08 d, when fed on *E. orientalis* and *E. sexmaculatus*, respectively. 

### 3.2. Age-Specific Survival Rate

When *N. barkeri* fed on *E. orientalis* or *T. putrescentiae*, all its immature survived (Table 2). When *N. barkeri* fed on *O. biharensisin*, mortality was observed at the deutonymph stage of this predatory mite, but not at the egg and other immature stages. The eggs of the predator fed on *E. sexmaculatus* survived, but an increasing mortality was observed during the protonymph (*F* = 1067.077; df1 = 3, df2 = 8; P < 0.001) and deutonymph (*F* = 1323.392; df1 = 3, df2 = 8; P < 0.001) stages. The accumulated survival rate of this predatory mite from egg to adult was 93.60% for those fed on *O. biharensisin* and 71.42% for those fed on *E. sexmaculatus* (*F* = 2101.222; df1 = 3, df2 = 8; P < 0.001).

### 3.3. Adult Longevity and Reproduction

**Figure 3:**
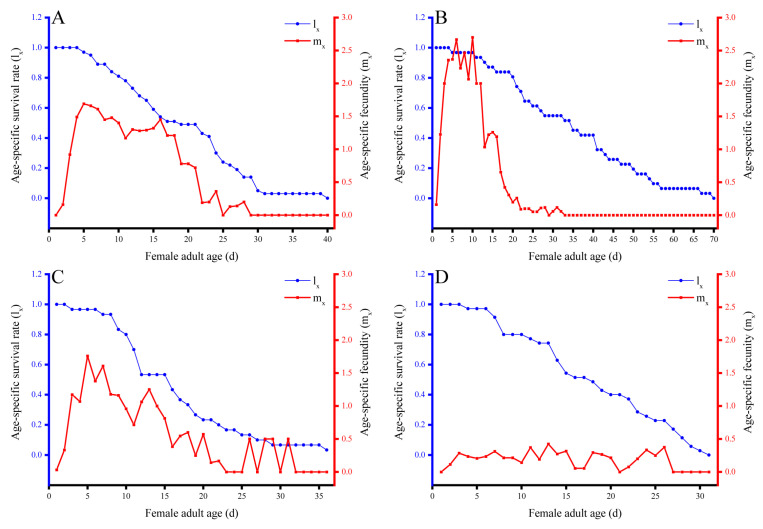
Age-specific survival rate (l_x_) and age-specific fecundity (m_x_) of *Neoseiulus barkeri* fed on different preys. (**A**) *Tyrophagus putrescentiae*, (**B**) *Eutetranychus orientalis*, (**C**) *Oligonychus biharensisin* and (**D**) *Eotetranychus sexmaculatus*. l_x_ is age-specific survival rate (proportion of individuals alive at age x), and m_x_ is age-specific fecundity (the number of female progenies per female at age x).

Preys had a notable impact on the longevity of female adult of *N. barkeri*. The longest recorded female adult longevity was observed in individuals fed on *E. orientalis* (34.55 ± 3.02 d), followed by those fed on *T. putrescentiae* (19.10 ± 1.41 d), both of which were significantly longer than those fed on *O. biharensisin* and *E. sexmaculatus* (approximately 15 d) (*F* = 26.441; df1 = 3, df2 = 138; P < 0.001) (Table 3). Additionally, the longest female adult age of *N. barkeri* was recorded in individuals fed on *E. orientalis* (69 d), followed by those fed on *T. putrescentiae* (39 d), *O. biharensisin* (36 d) and *E. sexmaculatus* (30 d) (Figure 3).

Different types of prey had significant effects on the reproductive parameters of *N. barkeri*. The pre-oviposition period was shortest when fed on *E. orientalis* (1.42 ± 0.21 d), followed by *T. putrescentiae* (3.45 ± 0.19 d) (*F* = 10.659; df1 = 3, df2 = 120; P < 0.001). Feeding on these two preys resulted in a longer oviposition period of approximately 13.5 d (Table 3) (*F* = 48.624; df1 = 3, df2 = 167; P < 0.001). In contrast, the longest pre-oviposition period and shortest oviposition period for *N. barkeri* were observed when fed on *E. sexmaculatus*. Regarding daily egg production, *N. barkeri* females fed on *E. sexmaculatus* did not show any peak, while peaks appeared in females fed on *E. orientalis*, *O. biharensisin* and *T. putrescentiae*. These peaks corresponded to 10 d, 5 d and 5 d after the emergence of adults (Figure 3). The maximum reproductive ability was found when *N. barkeri* fed on *E. sexmaculatus*, with eggs laid daily at 0.37 eggs per female per day. But the maximum reproductive ability of *N. barkeri* was recorded as 1.2 eggs per female per day for 15 d when fed on *T. putrescentiae*. Females of *N. barkeri* reared on *E. orientalis* had significantly greater fecundity, producing 29.35 ± 2.25 eggs per female, compared to those reared on other three preys (*F* = 102.457; df1 = 3, df2 = 168; P < 0.001) (Table 3). The fecundity of *N. barkeri* females fed on *E. sexmaculatus* was only 1.87 ± 0.50 eggs per female. As shown in Table 3, the sex ratios of *N. barkeri* fed on *E. sexmaculatus*, *E. orientalis* and *T. putrescentiae* were all female biased (*F* = 222.170; df1 = 3, df2 = 8; P < 0.001).

### 3.4. Life Table Parameters

Based on the developmental time, survivorship, fecundity and longevity of *N. barkeri* when fed on different preys, population life table parameters of this predator were analyzed and presented in Table 4. The results showed that the type of prey significantly influenced the growth of the population of *N. barkeri*. The net increase rate, intrinsic rate of increase, and finite rate of increase of *N. barkeri* reached their highest values of 29.650, 0.226 and 1.253, respectively, when the predator was fed with *E. orientalis*. Conversely, these parameters were lowest when *N. barkeri* were fed with *E. sexmaculatus* with values of 2.556, 0.041 and 1.042, respectively. When feeding on *O. biharensisin* and *T. putrescentiae*, the values of these parameters for *N. barkeri* are comparable. These results demonstrate that the population of *N. barkeri* had significantly better growth when fed with *E. orientalis*, whereas *E. sexmaculatus* had the most unfavorable impact on its population growth.

## 4. Discussion

Since *N. barkeri* can feed on a diverse range of species, such as spider mites, eriophyid mites, storage mites, broad mites, thrips, whitefly eggs, fungi and nematodes, and are easy to mass-produce, it has become an ideal biocontrol agent widely used to regulate populations of phytophagous pests in agricultural and ornamental crops [3,10,17,18]. In this study, we firstly evaluated the biological performance of *N. barkeri* against three phytophagous mites including *E. sexmaculatus*, *E. orientalis* and *O. biharensisin*, which cause serious damage on rubber trees in China [24,25]. The results indicate that *N. barkeri* is capable of completing its life cycle and successfully reproducing on these three mites. However, we observed developmental mortality in this predatory mite when fed on *E. sexmaculatus* and *O. biharensisin*, and a very low reproductive ability when fed on *E. sexmaculatus*.

The durations of *N. barkeri* larval and protonymphal development were found to differ significantly depending on the species of spider mites infesting rubber trees on which they preyed upon. Among the three different types of spider mites, the immature developmental period of *N. barkeri* was observed to be the shortest (around 5.5 d) when fed on *E. sexmaculatus* and *O. biharensisin*, comparable to or shorter than other prey mite species such as *Colomerus vitis* (5.43 d), *Aceria guerreronis* (5.6 d), *T. urticae* (8.45 d) and thrips (6.2 d) at 25 °C or higher temperatures, as evaluated until now [9,17,18,19,20,21]. When *N. barkeri* fed on *E. orientalis*, the immature development period and generation time of *N. barkeri* were the shortest found (5.25 d and 6.67 d, respectively), much lower than when compared to *T. putrescentiae*, a preferred prey used for mass-rearing *N. barkeri* along with two other studied preys [13,30].

The oviposition period, longevity and fecundity of female *N. barkeri* fed on *E. orientalis* and *O. biharensisin* fall within the range of data obtained when it fed on other preys, such as mites, thrips and whiteflies in previous studies [17,20]. However, when fed on *E. orientalis*, female *N. barkeri* exhibited significantly longer oviposition period, greater longevity and higher fecundity than those fed on *O. biharensisin*. Moreover, when compared to the factitious prey, *T. putrescentiae*, the oviposition period and fecundity of female *N. barkeri* when evaluated against *E. orientalis* were also much longer or higher. On the other hand, when the food source was *E. sexmaculatus*, the oviposition period and fecundity of female *N. barkeri* were significantly shortened and reduced compared to previous studies using other preys as food source (oviposition period ranged from 17.85 to 28.15 d, and fecundity ranged from 20.50 to 40.25 eggs per female) [9,17,18,19,20,21]. Interestingly, it has been observed that *N. barkeri* tends to prey on *E. sexmaculatus* more efficiently than on *E. orientalis*, *O. biharensisin* or *T. putrescentiae*, with the highest number of *E. sexmaculatus* consumed by *N. barkeri* within a day [29]. This suggests that the nutritional composition of *E. orientalis* is better suited for the development and reproduction of *N. barkeri* than that of *E. sexmaculatus*. The latter may contain harmful substances that negatively affects the fecundity of female *N. barkeri*.

Generally, the offspring sex ratio (percentage of females) of predatory phytoseiid mites is strongly biased towards female [20,33,34,35]. Consistent with previous literatures, the offspring sex ratios of *N. barkeri* fed on *E. sexmaculatus*, *E. orientalis* and *T. putrescentiae* showed a female bias. However, when this predatory mite was fed on *O. biharensisin*, it did not exhibit a preference for female progeny. The sex ratio of *N. barkeri* fed on *E. orientalis* was higher than those observed for mites fed on other species including the date dust mite *Oligonychus afrasiaticus* (66.6%), the tetranychid mite *Tetranychus urticae* (70%), the grape erineum mite *Colomerus vitis* (73.33%) and the citrus flat mite *Brevipalpus lewisi* (56.67%), for which the highest value reported is 80% [17,20,36]. It has been discovered that temperature can influence the sex ratio of *N. barkeri*, such that the percentage of females is highest at 35 °C and lowest at 25 °C when female predatory mites fed on *C. vitis*, *T. urticae* or *B. lewisi* [17]. Therefore, if *N. barkeri* were fed on *E. orientalis* at a higher temperature, it is probable that a larger percentage of female offspring would be produced.

Based on the life table parameters, it is evident that the growth of *N. barkeri* population is significantly affected by various preys. The population growth rates for *N. barkeri* fed on *E. orientalis* and *E. sexmaculatus* were found to be most promising and unfavorable, respectively. This can be substantiated by the intrinsic rate of natural increase (*r_m_*), which was 0.226 when fed on *E. orientalis*, while it was 0.041 on *E. sexmaculatus*. Notably, the intrinsic rate of natural increase (0.226) and net increase rate (*R*_0_) (29.65) of *N. barkeri* when fed on *E. orientalis* were much higher than those obtained on various preys at 25 °C or even higher temperatures as per previous studies [17,20]. For instance, high values of these two parameters in *N. barkeri* were evaluated in *Thrips tabaci* (*R*_0_ = 27.78, *r_m_* = 0.22) [8] and *T. urticae* (*R*_0_ = 22.02, *r_m_* = 0.22) [21] at 25 °C. Moreover, these two parameters observed in *N. barkeri* when fed on *O. biharensisin* were within the ranges (*R*_0_, 10.44–30.87, and *r_m_*, 0.09–0.27) previously reported on different preys [17,20], and are comparable to those recorded on *T. putrescentiae*. The mean generation time (*T*) showed the lowest value for *N. barkeri* when fed on *E. orientalis* compared to the values obtained in previous studies (12.26–19.76 d) [9,17,18,19,20,21]. These data indicate that *E. orientalis* is highly suitable for the population growth of *N. barkeri*.

According to the biological performance of *N. barkeri* when fed on *E. sexmaculatus*, *E. orientalis* and *O. biharensisin*, this predatory mite has promising potential in controlling of *E. orientalis* and *O. biharensisin* in the field, particularly with regard to protecting rubber trees. This is primarily due to its ability to thrive well on these two types of pests, as well as to complete its lifecycle and produce sufficient progeny that help support population growth. Considering the favorable promising immature development time, reproductive capability and population growth potential of *N. barkeri* on *E. orientalis*, it would be a favorable prey for mass-rearing *N. barkeri*. Although *N. barkeri* is not well-suited for establishing a population on *E. sexmaculatus*, it favors preying on this phytophagous mite [29]. Furthermore, *E. sexmaculatus*, *E. orientalis* and *O. biharensisin* are all phytophagous mites that damage rubber trees during the same time period [24,25,26]. Pollen grains of some plants in the rubber forest can serve as alternative food sources for *N. barkeri* to complete their life history and reproduce [19,36]. Therefore, *N. barkeri* can efficiently control *E. sexmaculatus* in the field when other available prey is present along with pollen food. Additionally, it is reasonable to assume that *N. barkeri* will perform better under natural environments with the presence of different spider mites and pollen food.

## 5. Conclusions

Our study represents the first attempt to evaluate the biological performance of the predatory mite *N. barkeri* in controlling spider mites including *E. sexmaculatus*, *E. orientalis* and *O. biharensisin* on rubber trees. Our results indicate that *N. barkeri* fed on *E. orientalis* and *O. biharensisin* yielded positive results, including high rates of developmental, fecundity and population parameters. These results exceeded or were comparable to data obtained using *T. putrescentiae* for mass-rearing *N. barkeri*. Although *N. barkeri* was unable to sustain its population on *E. sexmaculatus* due to its low reproductive rate, it exhibited considerable predation when consumed on this prey resulting in completing its life cycle. Our results underscore the promising potential of *N. barkeri* in controlling spider mites including *E. orientalis* and *O. biharensisin*, and possibly *E. sexmaculatus*, which could prevent substantial damage to rubber trees. Our study can serve as a guide for further investigations aimed at fully exploring the biocontrol potential and effectiveness of *N. barkeri* against spider mites on rubber trees, subsequently being used in augmentative release programs.

## Figures and Tables

**Table 1:** Development duration (days ± S.D.) of *Neoseiulus barkeri* fed on different preys.

Species	Egg	Larva	Protonymph	Deutonymph	Generation Time
*Tyrophagus putrescentiae*	1.65 ± 0.07 a	0.74 ± 0.04 bc	1.61 ± 0.10 a	1.79 ± 0.08 a	9.24 ± 0.06 b
*Eutetranychus orientalis*	1.67 ± 0.04 a	0.67 ± 0.03 c	1.57 ± 0.08 a	1.34 ± 0.09 b	6.67 ± 0.08 c
*Oligonychus biharensisin*	1.68 ± 0.07 a	1.00 ± 0.10 b	1.54 ± 0.23 a	1.09 ± 0.07 b	10.95 ± 1.25 a
*Eotetranychus sexmaculatus*	1.72 ± 0.06 a	1.52 ± 0.11 a	1.45 ± 0.11 a	1.25 ± 0.12 b	12.50 ± 0.08 a

The means within the same column followed by different letters are significantly different (*p* < 0.05).

**Table 2:** Survival rate (% ± S.D.) of immature stages of *Neoseiulus barkeri* fed on different preys.

Species	Egg	Larva	Protonymph	Deutonymph	Egg-Adult
*Tyrophagus putrescentiae*	100.00 ± 0.00 a	100.00 ± 0.00 a	100.00 ± 0.00 a	100.00 ± 0.00 a	100.00 ± 0.00 a
*Eutetranychus orientalis*	100.00 ± 0.00 a	100.00 ± 0.00 a	100.00 ± 0.00 a	100.00 ± 0.00 a	100.00 ± 0.00 a
*Oligonychus biharensisin*	100.00 ± 0.00 a	100.00 ± 0.00 a	100.00 ± 0.00 a	93.55 ± 1.82 b	93.60 ± 2.05 b
*Eotetranychus sexmaculatus*	100.00 ± 0.00 a	100.00 ± 0.00 a	93.20 ± 3.05 b	76.81 ± 4.01 c	71.42 ± 4.30 c

The means within the same column followed by different letters are significantly different (*p* < 0.05).

**Table 3:** Duration of adult (days ± S.D.), fecundity (eggs/female) and sex ratio (female:male) of *Neoseiulus barkeri* fed on different preys.

Species	Pre-Oviposition Period	Oviposition Period	Longevity	Fecundity	Sex Ratio
*Tyrophagus putrescentiae*	3.45 ± 0.19 b	13.64 ± 10.2 a	19.10 ± 1.41 b	18.45 ± 1.62 b	1.95 ± 0.02 b
*Eutetranychus orientalis*	1.42 ± 0.21 c	13.32 ± 0.80 a	34.55 ± 3.02 a	29.35 ± 2.25 a	2.10 ± 0.06 a
*Oligonychus biharensisin*	5.64 ± 0.84 a	7.65 ± 0.60 b	14.71 ± 1.26 c	15.36 ± 1.11 c	1.00 ± 0.00 d
*Eotetranychus sexmaculatus*	6.56 ± 1.26 a	2.22 ± 0.65 c	14.88 ± 1.32 c	1.87 ± 0.50 d	1.70 ± 0.02 c

The means within the same column followed by different letters are significantly different (*p* < 0.05).

**Table 4:** Life table parameters of *Neoseiulus barkeri* fed on different preys.

Species	*R*_0_	*T*	*r*_m_	*λ*	DT
*Tyrophagus putrescentiae*	12.390	15.180	0.166	1.181	4.176
*Eutetranychus orientalis*	29.650	15.020	0.226	1.253	3.072
*Oligonychus biharensisin*	12.772	18.677	0.136	1.146	5.082
*Eotetranychus sexmaculatus*	2.556	22.944	0.041	1.042	16.947

*R*_0_, net increase rate; *T*, mean generation time; *r*_m_, intrinsic rate of increase; *λ*, finite rate of increase; DT, doubling time.

## Data Availability

All the information is in this document.
